# Intelligent Method for Real-Time Portable EEG Artifact Annotation in Semiconstrained Environment Based on Computer Vision

**DOI:** 10.1155/2022/9590411

**Published:** 2022-02-12

**Authors:** Xuesheng Qian, Mianjie Wang, Xinyue Wang, Yihang Wang, Weihui Dai

**Affiliations:** ^1^Institute of Systems Engineering and Collaborative Laboratory for Intelligent Science and Systems, Macau University of Science and Technology, Macao 999078, China; ^2^School of Management, Fudan University, Shanghai 200433, China; ^3^Shanghai Ineutech Techonolgy Co., Ltd., Shanghai 200072, China; ^4^College of Letters and Science, University of California, Berkeley, CA 94720, USA; ^5^Steinhardt School of Culture, Education, and Human Development, New York University, New York, NY 10003, USA

## Abstract

As a convenient device for observing neural activity in the natural environment, portable EEG technology (PEEGT) has an extensive prospect in expanding neuroscience research into natural applications. However, unlike in the laboratory environment, PEEGT is usually applied in a semiconstrained environment, including management and engineering, generating much more artifacts caused by the subjects' activities. Due to the limitations of existing artifacts annotation, the problem limits PEEGT to take advantage of portability and low-test cost, which is a crucial obstacle for the potential application of PEEGT in the natural environment. This paper proposes an intelligent method to identify two leading antecedent causes of EEG artifacts, participant's blinks and head movements, and annotate the time segments of artifacts in real time based on computer vision (CV). Furthermore, it changes the original postprocessing mode based on artifact signal recognition to the preprocessing mode based on artifact behavior recognition by the CV method. Through a comparative experiment with three artifacts mark operators and the CV method, we verify the effectiveness of the method, which lays a foundation for accurate artifact removal in real time in the next step. It enlightens us on how to adopt computer technology to conduct large-scale neurotesting in a natural semiconstrained environment outside the laboratory without expensive laboratory equipment or high manual costs.

## 1. Introduction

Electroencephalography (EEG) has been proved to be a useful methodological tool for understanding brain activities, including the processes of perception, cognition, and decision, which are the basis of daily behaviors, business, and engineering activities.

With the great attention to human decision-making and the recognition of limitations of traditional psychological/self-reported driven approaches [1–4], the neuromanagement on revealing the mechanism of human's behavior and decision-making based on brain imaging technology is promoted [5–7]. However, due to the high cost of purchasing and maintaining neurometric equipment and the complex operation and data analysis mode, brain technologies are limited to the laboratory environments and hindered from becoming widespread.

Neurophysiological measurements initially rely on high-cost equipment, complex systems, and many professional operations (e.g., measuring the size of the head, marking the position of electrodes on the scalp, placing electrodes on the scalp, and using conductive glue). Benefiting from the development of portable EEG technology (PEEGT), devices become cheaper and smaller, such as single-electrode NeuroSky MindWave, four-electrode Muse, and fourteen-electrode Emotiv. What is more, with simple preparation, EEG data are collected through the wireless network. The PEEGT devices are suitable for nonclinical studies with better interactive experiences [8]. Nowadays, more and more research and commercial applications use PEEGT as a measurement tool. The utilization of PEEGT significantly expands the application of neurophysiological measurements and dramatically increases the practicability of neurometric equipment, such as in marketing [5], management [9], education [10], and engineering [11].

The PEEGT effectively reduces the threshold of the experimental environment in business and management situations. It is especially suitable for the volatile, uncertain, complex, and ambiguous (VUCA) environment. It makes large-scale and long-time neurophysiological measurements at the lowest cost possible. Moreover, subjects are allowed to have slight movements in position during a long-lasting experiment, for example, adjusting sitting or head posture like in a natural situation; we called this the semiconstrained environment. The semiconstrained environment is different from a constrained environment where subjects in the laboratory are under strict restrictions on autonomous activities. There is also a distinction between a semiconstrained and unconstrained environment where subjects have significant activity freedom. Nevertheless, fewer experimental restrictions in semiconstrained and unconstrained environments bring more artifacts by physical activities, which is difficult for artifacts operation.

Annotation of artifacts is a prerequisite for removing artifacts and EEG analysis. Existing methods of artifact annotation face the tradeoff between testing convenience and annotation accuracy, making it inapplicable in daily business scenarios where there are high requirements for both the convenience of collection and the accuracy of artifact annotation. The methods relying on additional reference signals or biological signal equipment are not applicable for the daily context that needs PEEGT. In contrast, methods relying on multiple algorithms have a long computing delay and are less accurate than methods with additional reference signals. Last but not least, most existing methods can only be used in postacquisition or offline settings, but real time is an essential demand of artifact annotation in business scenarios.

Considering existing methods, effectively annotating artifacts generated from physical activities usually requires manually annotating artifacts in postacquisition offline settings, which is extremely time-consuming and dependent on the data operator's expertise level. What is worse, to ensure the accuracy of the testing results and meet the requirements of business scenarios, tests with PEEGT usually need a larger sample size, a longer test time, and a more uncertain test environment. In a word, it is a great challenge to apply neuroscience in scenarios emphasizing “natural” due to the lack of adequate and suitable artifact processing technology. The artifact problem limits PEEGT to take advantage of portability and low test cost, which is a crucial obstacle for the potential application of PEEGT in the natural environment. It is worth noting that a challenge also exists in the laboratory EEG test, but the traditional experiment has more reference equipment assistance and more processing time. Therefore, in most laboratory cases, the limitation can be arranged by investing more resources.

Intelligent algorithms in computer vision (CV) bring new possibilities to solve the above difficulties. This article proposes one intelligent computing method on real-time portable EEG artifact annotations with computer vision, which changes the original artifact postprocessing mode based on signal recognition to the artifact preprocessing mode based on behavior recognition. It is especially suitable for artifact problems in semiconstrained environments involved in most real scenes in engineering and management. Besides, it provides the foundation for the subsequent artifact intelligent removal by the machine learning algorithm.

Our main contributions can be summarized as follows:

1. We introduce a thought about changing the original signal recognition-based artifact postprocessing mode to the artifact preprocessing mode based on behavior recognition by the computer algorithm, making it possible to process artifacts in real time using only a camera instead of additional expensive neurological equipment and amounts of manual processing.
2. We propose an intelligence method based on computer vision to automatically annotate the time segments and categories of artifacts caused by blinks and head movements in real time, which is of great importance for the large-scale application and real-time analysis of PEEGT and traditional EEG.
3. We suggest a procedure about how to use large-scale neurological measurement in business and management scenarios with fewer restrictions on subjects' physical activities. The method ensures that the artifacts caused by the physical activities in blinks and head movements can be annotated in real time, which greatly expand neuroscience research into real applications environment including engineering and management.

## 2. Literature

### 2.1. EEG Artifacts

Electroencephalogram (EEG) is a standard method of measuring human brain activities that change over time in the form of electrograms. EEG data has shown great potential in research and commercial applications. It can be used as a diagnostic and monitoring tool for clinical applications, such as quantifying anesthesia levels before and during surgery [12], and film and advertising evaluation, such as film and television effect research [13]. However, there are many inherent challenges in EEG analysis, specifically the removal of various artifacts.

Generally, the artifacts of the EEG signal are mainly from the physiological activities of the participant and the additional noise artifacts introduced by the EEG acquisition instrument. The latter can be reduced or even eliminated by improving the operating performance of the EEG signal acquisition instrument. However, bioelectrical signals generated by physiological activities are inevitably hidden in the EEG signals or even submerged in the EEG signals, which seriously affects the authenticity of the EEG signals and complicates the research work of feature extraction and EEG signal analysis. Blinks and head movements are two kinds of major signal artifacts. As a result, the research work of feature extraction and analysis of the EEG signals becomes complicated.

**Figure 1:**
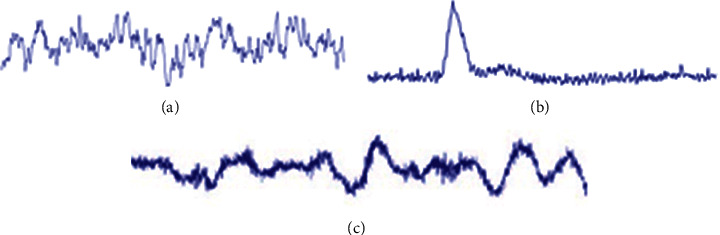
Typical artifact waveforms [14, 15]. (a) Clean EEG. (b) Blink artifact. (c) Head movement artifact.

Figure 1 [14, 15] shows typical artifact waveforms with obvious and common interference to EEG signals. The blink artifact is generated by blinking eyes, while the head movement artifact is generated by head rotation or movements.

### 2.2. EEG Artifacts Annotation Methods

EEG artifact annotation has always been a challenge in the EEG signals analysis process. Challenges come from the complexity of the method and the nonlinearity of the noise. For example, due to the “nonlinear” nature of the artifact, it is difficult to annotate the artifacts from the original EEG data without affecting the normal signal. In addition, some methods cannot be used for real-time applications. Until now, although researchers have been exploring lots of EEG artifact annotation methods, there is still no consensus on which algorithm is most suitable for a specific application.

In general, there are two categories of EEG artifact annotation methods: direct labeling of artifacts and indirect separation of artifacts [16].

#### 2.2.1. Direct Labeling of Artifacts

Direct labeling of artifacts refers to annotating artifact signals in real time when EEG signal is collected. However, it requires additional reference signals from reference electrodes channels or additional biological monitoring equipment. Li et al. [17] adopted additional channels of real EMG from neck and head muscles as input and realized the significant separation of EEG and EMG artifacts without losing the underlying EEG features. Mannan et al. [18] realized the simultaneous collection of EEG and EOG signals by adding the channels of EOG electrodes and combined independent component analysis (ICA), regression, and high-order statistics to identify and eliminate artifactual activities from EEG data. In terms of adding monitoring equipment, König et al. [19] used a laboratory-level eye tracker to annotate blink and eye movement artifacts in the constrained environment. Compared with the traditional manual artifact annotation, adding reference equipment has dramatically improved the accuracy and efficiency. Nevertheless, additional channels cause the additional possibility of artifacts. On the other hand, the additional expensive and complicated laboratory equipment will increase the burden of operators and participants and the terms of the test environment.

#### 2.2.2. Indirect Labeling of Artifacts

Indirect separation of artifacts refers to separating the mixed signals by multiple integrated algorithms without the reference electrodes or monitoring equipment. Jan et al. [20] improved the ICA method to better artifact removal. Chang et al. [21] used artifact subspace reconstruction (ASR) to preprocess EEG data and, combined with the ICA separation method, greatly improved the accuracy of artifact removal. Indirect separation of artifacts avoids extra electrodes, making it convenient in the test environment and reducing the extra noise. However, the method needs to integrate a variety of algorithms, increasing the algorithm's complexity and reducing the artifact annotation's real-time performance. Moreover, due to the lack of reference supervision of the artifact signals, the accuracy of the indirect artifact annotation is generally lower than those of the direct methods.

Although the two above-mentioned methods for artifact annotation have made explorations from different directions and achieved good results, they are based on sacrificing one aspect to improve the other, making it hard to apply in daily business scenarios. More specifically, the direct labeling of artifacts achieves better accuracy of artifact annotation with additional reference electrodes and laboratory equipment at the cost of the convenience in equipment operation and the comfort of participants. In contrast, the method of indirect separation of artifacts achieves more convenient signal acquisition with integrated algorithms at the cost of the accuracy of artifact annotation. Because the scenarios of daily business have high requirements for both the convenience in signal acquisition and the accuracy of artifact annotation, methods used in these scenarios should not only simplify the test environment and ease participant's test burden but also ensure the accuracy of artifact annotation and take the real-time requirements into account.

### 2.3. Semiconstrained Environment

In neuroscience, research and experiments are conducted in two kinds of settings, laboratory settings and nonlaboratory settings. A laboratory test environment is carefully designed, in which researchers and participants need to follow strict restrictions and guidelines. Usually, other experimental settings outside laboratories are nonlaboratory settings. However, in most of the research articles, researchers either directly employ the term without further clarification [22] or use nonlaboratory settings to refer to a relatively broad idea [23]. For example, experiments conducted in the unattended home [24] and observational studies conducted in a community [25] are different in settings, but both are referred to as “nonlaboratory.”

In the past literature, scientists did not specify how to set up neurological devices in conditions other than a laboratory. It is an unexpected gap in neural experiments. In many cases, it is unrealistic to keep participants motionless in a place with no distraction, especially in the natural environment using PEEGT. Therefore, to provide a more precise scope to clarify the application of our methods and algorithms, we define a term in our paper, a “semiconstrained environment,” compared to a fully attended laboratory and an unattended natural setting. A semiconstrained environment describes an experimental setting where participants are required to wear a device; however, the research does not propose a highly restricted demand on participants (such as zero movements at all during measurement) since it is not always available to set up a lab-based environment.

## 3. Methodology

### 3.1. Method Proposed

In traditional EEG signal artifact processing without additional reference equipment, experienced signal processors must manually mark the abnormal signal period in the test signal after completing signal preprocessing, such as band-pass filtering. Usually, such an operation needs to be repeated 10–20 times for each subject, and more marks lead to a better operation effect. Then, the unique signal processing software will use algorithms such as ICA to automatically remove similar artifact signals according to the manually marked signal form. This method requires a long time and high personnel investment. In principle, such a manual postmarking and removal algorithm cannot ensure accuracy, and there is a certain degree of fuzzy space, even if it is existing in common practice.

**Figure 2:**
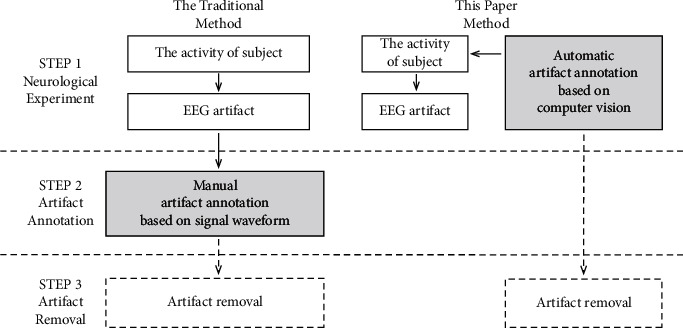
The proposed method.

In this paper, CV is introduced into neurological experiments. It can detect subjects' behaviors simultaneously during the experiment and mark the events that may produce artifacts from the source shown in Figure 2. Thus, it improves the real-time performance of artifact processing, avoids the later manual investment, and makes artifact marking no longer an ambiguous activity based on experience.

### 3.2. Process

**Figure 3:**
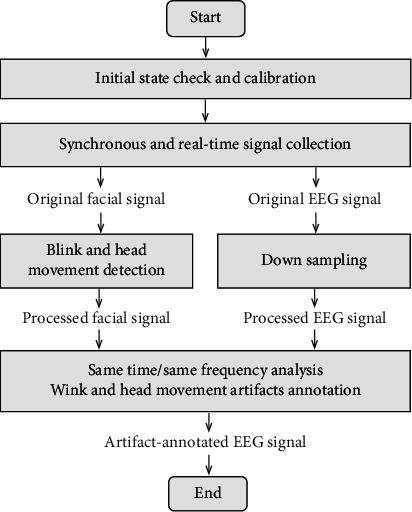
Method of EEG artifact annotation with supervised computer vision.

The key to accurate artifact annotation in a semiconstrained environment is timely identifying the most common participants' physical activities that may cause artifacts, for example, blinks and head movements. In this paper, a method for annotating blink and head movement artifacts with computer vision in daily business scenarios is proposed, which meets requirements under this semiconstrained environment to a great extent. The method is shown in Figure 3. Firstly, it is necessary to collect the participants' initial state and calibrate the algorithm. Specifically, the subjects' eye-closing threshold is collected to measure blinking state during the test, and the subjects' initial sitting orientation is for the measurement of head movement state. Notably, the initialization of PEEGT equipment and standard commercial high-definition cameras, unlike the cumbersome operation of lab eye-tracking equipment, performs the initial state check and calibration only to ensure the equipment availability and to collect the initial eye and head positions of the subjects.

Next, the PEEGT equipment and camera are used to synchronously collect participants' facial signals and EEG signals in real time. The blinks and head movements are detected with computer vision based on facial feature points. It is critical to note that the original EEG signals are downsampled in sync with the facial signal. Finally, the facial and the EEG signals in the same time series are analyzed and processed with the same analysis frequency. The EEG signals with blink and head movement artifacts are annotated.

**Figure 4:**
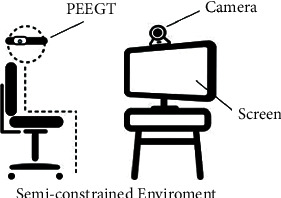
The site-setting of blink and head movement artifacts annotation with supervised computer vision.

The site-setting of the method is shown in Figure 4; the participant is wearing PEEGT devices and looking at the screen. In a semiconstrained environment, the participant can adjust his or her posture during the experiment. The camera ensures that head activity can be entirely recorded. Based on the computer algorithm with supervised real time and synchronization, the method can annotate the time segments of blinks and head movements that cause EEG artifacts.

### 3.3. Recognition Algorithm

#### 3.3.1. Facial Feature Points Positioning

As mentioned above, the recognition algorithm first needs to collect the participant's facial signals and then monitor facial condition according to the specific facial features. The purpose of facial feature points positioning is to further define facial feature points (facial features and edge). The algorithms collect the baseline values of participants before the test, and the collected data and standards are unified. Therefore, the judgment threshold has corresponding calculations and standards for people of different face types.

The methods of facial feature point positioning can be categorized into the global method, the constrained local model (CLM) method, and the regression method, based on detecting face appearance and face shape information. The global method is to model the global face appearance and global face shape information explicitly [26]. The CLM explicitly models the local face appearance and the global face edge information [27]. The regression method uses global and local appearance information to implicitly embed global shape information for joint feature point detection [28]. Generally, the regression method performs better because it contains more information compared to the other two. One representative algorithm of the regression method is Ensemble of Regression Trees (ERT) [29]. ERT is often used in facial feature point positioning because it is swift (it takes about 1 ms to detect each facial feature point) and can deal with the missing calibration of some key points in the training set.

**Figure 5:**
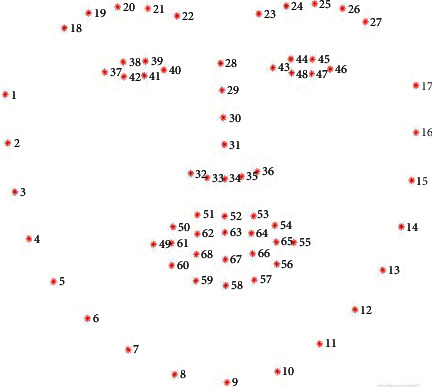
68 key feature points of each face.

The method in this paper uses the ERT algorithm to position 68 key feature points (as shown in Figure 5) of each face within 1 ms through three steps: shape invariant split tests, choosing the node splits, and feature selection proposed by Kazemi and Sullivan [29], which can estimate the face's landmark positions directly from a sparse subset of pixel intensities, achieving super-real-time performance with high-quality predictions. The pseudocode for this program is as follows (Algorithm 1).

  def face_landmarks ():
    predictor = dlib initializes shape_predictor (“shape_predictor_68_face_landmarks.dat”)
    # cv2 is the OpenCv library
    cap = cv2 gets the first camera of the machine
    while (cap is opened):
      flag, im_rd = cap.read () builds 3D matrix
      img_gray = cv2.cvtColor (im_rd, cv2.COLOR_RGB2GRAY)
      faces = detector (img_gray)
      for *k*, *d* in enumerate (faces):
        shape = predictor (im_rd, *d*)
  END face_landmarks

**Figure 6:**
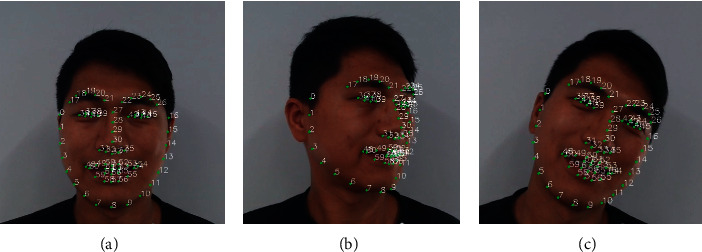
The implementation display of recognition effect.

The implementation display of the recognition effect in the practical example of this method is shown in Figure 6.

#### 3.3.2. Blink and Head Movement Detection

**Figure 7:**
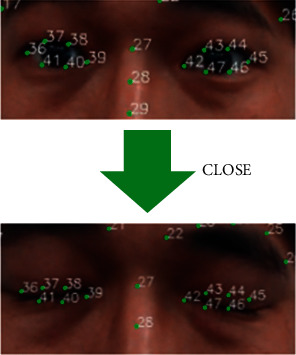
Method of blink detection.

*(1) Blink Detection*. In Subsection 3.3.1, basic facial information and 68 key feature points of each face are positioned. In the method proposed, a participant's blink is detected by analyzing the closure degree of the eye region (six feature points forming a closed ellipse), as shown in Figure 7.


(1) $$\begin{array}{c} \begin{array}{c} {\text{EAR}}_{L}=\frac{\left|\left|p_{37}-p_{41}\right|\right|+\left|\left|p_{38}-p_{40}\right|\right|}{2\left|\left|p_{36}-p_{39}\right|\right|},\\ {\text{EAR}}_{R}=\frac{\left|\left|p_{43}-p_{47}\right|\right|+\left|\left|p_{44}-p_{46}\right|\right|}{2\left|\left|p_{42}-p_{45}\right|\right|}. \end{array} \end{array}$$

Eye Aspect Ratio (EAR) is equal to the sum of the lengths of two vertical line segments divided by the double length of horizontal line segments. The EAR stands for the state of eyes' opening and closing, and *p*_*i*_ is one of the 68 feature points forming the eye region (as shown in formula (1)). Research shows that EAR can approach zero at the moment of closing eyes and return to the original value when opening eyes [30]. By monitoring whether the value of EAR fluctuates rapidly and approaches zero in real time, this method can identify whether the participant's eyes are closed. However, the threshold for blink detection is undefined in this method, introducing noises in the natural environment. Therefore, considering the sampling rate of CV, this paper monitors the blink state by setting the base value before measurement. That is, the data of eye-closed state for 1 minute before the test are collected, and the blink detection threshold Blink_threshold_ based on its average value is defined:

(2) $$\begin{array}{c} \begin{array}{c} {\text{Blink}}_{\text{threshold}}=\frac{1}{N}\overset{N}{\underset{i=1}{\sum }}B_{i} \end{array}. \end{array}$$

In the above equation, *N* represents the number of samples in 1 minute. Since the sampling rate of CV is 25, *N* = 1500. *B*_*i*_ is the data of eye-closed state collected at the *i*^th^ time. After collecting and calculating the blink detection threshold Blink_threshold_, blinks are detected according to the value:

(3) $$\begin{array}{c} {\text{EAR}}_{\text{per\_second}}=\min\left({\text{EAR}}_{p}\right),\\ {\text{Blink}}_{\text{status}}=\left\{\begin{array}{c} 1,\text{ if}\;{\text{ EAR}}_{\text{per\_second}}\leq \;{\text{Blink}}_{\text{threshold}},\\ 0,\text{ if }\;{\text{EAR}}_{\text{per\_second}}>\;{\text{ Blink}}_{\text{threshold}}, \end{array}\right) \end{array}$$

where EAR_per_second_ is the minimum value of EAR in each cycle. If EAR_per_second_ is less than or equal to Blink_threshold_, it is determined that there is blinking action within 1 second. If EAR_per_second_ is greater than Blink_threshold_, it is determined that there is no blinking action within 1 second. The pseudocode for this program is as follows (Algorithm 2).

  def face_ear ():
    if obtain 68 feature points of the face:
      ear_r_list.append (ear_r), ear_l_list.append (ear_l)
      ear_r_status, ear_l_status = 0
      If *N* = = 25:
        ear_r_persecond = min(ear_r_list), ear_l_persecond = min(ear_l_list)
        ear_r_list.clear, ear_l_list.clear
        If ear_r_persecond ≤  blink_r_threshold:
          ear_r_status = 1
        else:
          ear_r_status = 0
        If ear_l_ persecond ≤  blink_l_threshold:
          ear_l_status = 1
        else:
          ear_l_status = 0
       return ear_r_status, ear_l_status
     else return null
  END face_ ear

*(2) Head Movement Detection*. Head movement (HM) detection is related to face orientation. In facial feature point positioning, 68 key feature points, including eyes and nose, are extracted from each facial image. In this paper, we refer to the center points of the eyes and nose to define the face orientation coordinates [31].

**Figure 8:**
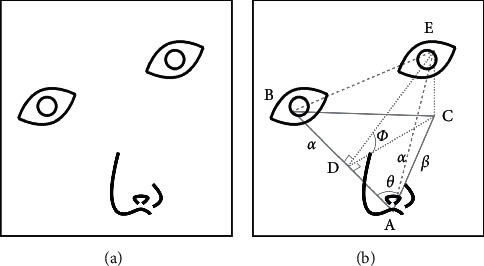
Facial image (a) and projection isosceles triangle model (b).

In (2), the eyes and nose have been correctly positioned. The three facial feature points determine an isosceles triangle by connecting lines between the three points. Considering the symmetry of the face, we can calculate the angle between the plane of the isosceles triangle and the image plane to determine the gaze direction. If one side of the triangle is located on the image plane, it is easy to calculate the angle of the gaze direction. The judgment of facial orientation is realized through the calculation of trigonometric function, as shown in Figure 8.

The triangle ABC is the projection of the isosceles triangle *ABE* on the image plane, which means that if the person in the picture looks straight ahead, the projection triangle will coincide with the isosceles triangle. The triangle *CDE* is located on the plane perpendicular to the image plane and isosceles triangle plane. If *θ* is the angle between line *α* and line *β* and *ϕ* is the angle between the image plane and the isosceles triangle plane, then

(4) $$\begin{array}{c} \cos\;\phi =\frac{\left|CD\right|}{\left|DE\right|},\\ \left|CD\right|=\left|AC\right|\sin\;\theta ,\left|AD\right|\\ =\left|AC\right|\cos\;\theta , \end{array}$$

cos *ϕ* can be calculated by trigonometric function as follows:

(5) $$\begin{array}{c} \begin{array}{c} \cos\;\phi =\frac{\left|AC\right|\sin\;\theta }{\sqrt{{\left|AB\right|}^{2}-{\left|AC\right|}^{2}{\text{cos}}^{2}\theta }} \end{array}. \end{array}$$

Therefore, determining the direction of the sight is to see how the isosceles triangle is projected on the image plane: the maximum distance from the eye to the mouth reveals the direction of the human gaze. After obtaining the participant's gaze direction, the attention direction is recorded, while the lateral (HM) of the participant is identified according to point *A*'s coordinates.

Similarly, for head nodding, it is calculated as follows:

(6) $$\begin{array}{c} d=\left|\left|p_{27}-p_{30}\right|\right|. \end{array}$$

*d*_*i*_ is obtained for each segment of detection. Thus,

(7) $$\begin{array}{c} D^{\prime }=\text{Median}\left\{d_{1},d_{2},d_{3},\ldots ,d_{N}\right\}. \end{array}$$

*N* = 1500 represents the number of samples in 1 minute. Then,

(8) $$\begin{array}{c} \phi^{\prime }=\frac{\left||D-D^{\prime }|\right|}{D^{\prime }}*90. \end{array}$$

In the above equation, *ϕ*′ is the angle at which the subject's head is nodding, ranging from 0 to 90°.

In addition, participants sit on the designated spot during the test in a semiconstrained environment and cannot move back and forth smoothly. Therefore, the above two detection conditions can meet the detection marks of most HM artifacts, and the HM can be estimated by the angle difference between the two moments.

Instead of judging the absolute HM angle of the participant, the proposed method calculates the relative HM angle change in EEG artifact annotation.

Therefore, it is necessary to have

(9) $$\begin{array}{c} \psi =\frac{1}{N}\overset{N}{\underset{i=1}{\sum }}\emptyset_{i},\\ \emptyset_{\text{relative}}=\psi_{t}-\psi_{t-1},\\ {\text{Head}}_{\text{status}}=\left\{\begin{array}{c} 0,\emptyset_{\text{relative}}\leq 10^{{}^{\circ}},\\ {1,10}^{{}^{\circ}}<\emptyset_{\text{relative }}\leq 30^{{}^{\circ}},\\ 2,\;\emptyset_{\text{relative}}>30^{{}^{\circ}},\; \end{array}\right) \end{array}$$

where *ψ* is the real-time HM angle of the participant relative to the right ahead direction in a sampling period. ∅_relative_ refers to the change of the HM angle of the participant in the current second relative to the previous second. If ∅_relative_ is less than 10°, it is judged to be relatively static and counted as 0. If ∅_relative_ is greater than 10° and less than 30°, it is judged as a micro-HM and counted as 1. If ∅_relative_ is greater than 30°, it is judged as a distinct HM and counted as 2. Head vertical and horizontal movements are calculated separately and Head_status_ depends on the bigger one. Therefore, HM artifacts are detected and marked as different levels in the EEG artifact annotation.

The pseudocode for this program is as follows (Algorithm 3).

  def face_angle ():
     if obtain 68 feature points of the face:
       head_angle_list.append (angle)
       current_angle = angle
       If *N* = = 25:
         head_change = current_angle–last_angle
         If head_change ≤ 10:
           head_status = 0
         else if 10 < head_change ≤ 30:
           head_status = 1
         else:
           head_status = 2
         head_angle_list.clear
         return head_status
       else:
         last_angle = angle
     else:
       return null
  END face_ angle

### 3.4. EEG Artifact Annotation with Supervised Computer Vision

**Figure 9:**
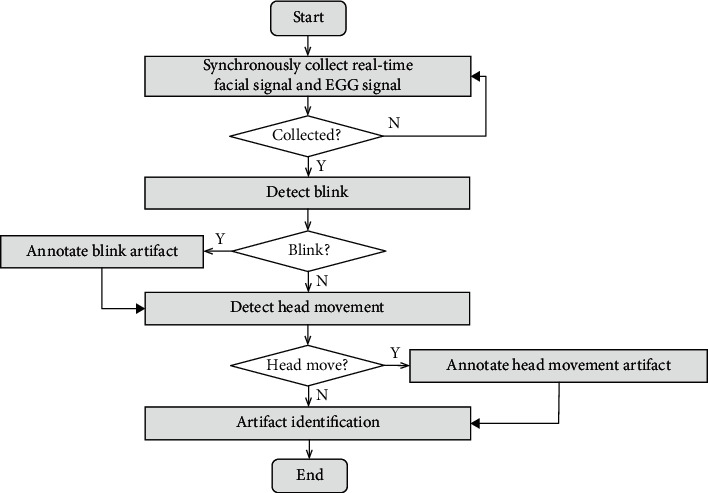
Process of EEG artifacts annotation with supervised computer vision.

The process of blink and head movement artifacts annotation with supervised computer vision is shown in Figure 9. Firstly, the participant's facial and EEG signals are collected synchronously and in real time. Then, the blink detection algorithm is used to determine whether the blink action occurs. If there is a blink, the EEG signal in this state is annotated. Then the head movement algorithm, which determines whether a head movement occurs, is activated. If there is no head movement, the blink artifact is identified. If there is a head movement, the EEG signal in this state is annotated, and the head movement and blink artifacts are both identified.

It should be noted that the time window of the above process is 1 second. In a semiconstrained environment where tests generally take a long time, the 1-second time window can significantly reduce the time complexity and space complexity of data processing if enough EEG signals are retained and improve the efficiency of the whole test process. Real-time feedback also plays an important role in meeting the diverse business application needs.

### 3.5. Method Implementation

Firstly, in algorithm implementation, we use Python 3.0 as the primary development language, and the development tool is PyCharm. The tool libraries used in the system development are Dlib, OpenCV, math, and NumPy. Specifically, Dlib is mainly used for face recognition and feature point labeling, OpenCV is mainly used for image processing and generation, math is used for mathematical algorithm calculation, and NumPy is used for feature point data processing.

Through the implementation, we verify the feasibility of the above method. In the example, we set the test environment and CV systems as in Figure 4 and performed the EEG acquisition for 24 seconds. In order to ensure the sensitive and accurate acquisition of EEG signals, we used the laboratory EEG equipment (ANT eego^TM^mylab) instead of PEEGT to measure the example.

**Figure 10:**
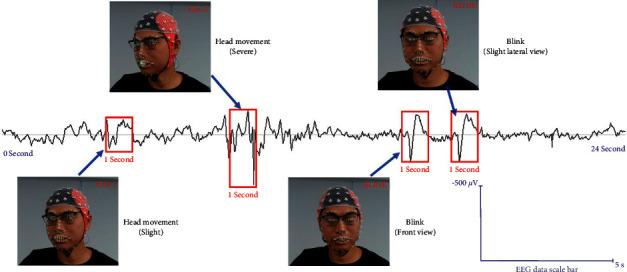
The example of the method implementation.

The model (by the author team) carried out four activities: blinking twice, towards two directions, and head movements twice, once slight and once severe. The implementation results indicated that all the activities were captured with the methods. All the artifacts were marked simultaneously by the 1-second time window, as demonstrated in Figure 10.

## 4. Experiment

### 4.1. Experiment Design and Participant

We designed the experiment to verify whether the proposed intelligent computing method effectively recognizes and annotates the subject's activities in an authentic test environment.

**Figure 11:**
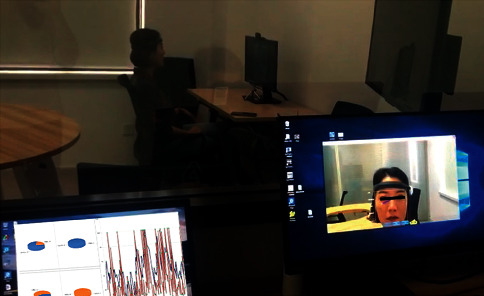
The experiment scene.

The experiment recruited one participant that watched TV reality show programs for 15 minutes wearing PEEGT equipment and the test site-setting as in Figure 4. In the semiconstrained environment, the participant was not told the requirement of body movement restriction, and no one else was present during the experiment. The camera above the screen recorded the participant's head movements and blinks. The experimenters were observed through one-way glass and real-time system data. The scene picture is shown in Figure 11.

Three observers with EEG artifact processing experience watched the recorded video. They manually marked the participant's activity in the video, including blinks (left eye, right eye, and two eyes) and head movements (slight: rotation or tilt more than 10° and less than 30°; severe: rotation or tilt more than 30°). To simulate a large-scale artifact process task, the three observers perform a 30-minute irrelevant annotating task before the labeling task of this experiment. The participant's video is played at normal speed during the annotating process, and backward progress is not allowed.

The experimental team also manually marked the participant's movements as the reference value to ensure the accuracy of the marking. If necessary, the video can play slowly and repeatedly. For operability, the time granularity of all manual marking is 1 second.

### 4.2. Experiment Result

**Figure 12:**
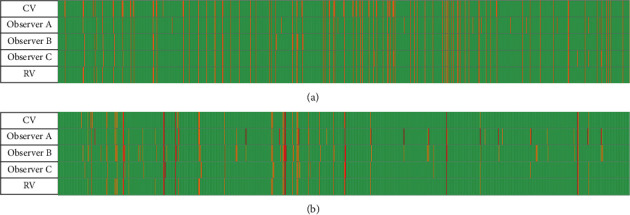
The original results of the experiment.

The activity markers of the participant were compared among the CV, three observers, and the reference value (RV, by experiment team). The original mark results are shown in Figure 12. The statistical result is shown in Table 1. The time granularity of all manual marking is 1 second, so the total represents 900 seconds, and each row has 900 horizontal grids in Figure 12. Note that the blinks here only refer to the ones detected by naked eyes.

## 5. Discussion

Overall, the experimental results show that the CV method in this paper got an ideal achievement, including sensitivity, specificity, and detection number. Moreover, the CV method has the advantages of real time and low cost.

For blink annotation, in general, the frequency of blinks correctly identified by the CV method is higher than that by the manual method, and the frequency of missed detections is lower under the close sensitivity and specificity. It is difficult for manual marking to maintain a high concentration and to notice the instant blink event for a long time, even with relevant data processing experience. In addition, the fuzziness of the human brain in judging events in unstructured data such as video will also lead to errors and omissions. The above reasons explain why the traditional artifacts processing cannot be applied to the business large-scale and long-time semiconstrained environment of PEEGT.

On the other hand, as for the CV method used in this experiment, the frequency of errors is slightly higher than that in the manual method in blink detection, which is due to categorizing the subject's eye-drooping activity as a blink event. From the perspective of bioelectrical signal interference, blinks and eye-drooping are the same. However, it is shown that even if the facial feature points can be captured all the time, the CV still has the possibility of recognizing some activity events incorrectly. Nevertheless, by optimizing the model, the false detection rate can be controlled, fully competent for the artifacts annotating long-time continuous EEG signal acquisition. In addition, there may be subtle differences between frequencies of two eyes' blinks in the CV method that independently detects binocular activity, which is distinguished from the observer's overall observation style. Thus the difference may be more significant for participants with greater eye size differences. However, in large-scale and long-term tests, the influence of the above slight differences is almost negligible, especially in a semiconstrained test environment.

For head movements, the results are similar to the blinks. The CV recognition shows a significant advantage in efficiency, effectiveness, sensitivity, and specificity close to manual marking. Then it shows the great advantage of intelligent computing in a long-term mechanical task, and the method proposed in this paper is effective.

## 6. Conclusion and Future Work

The method proposed by this paper changed the original artifact postprocessing mode based on signal recognition to the artifact preprocessing mode based on behavior recognition by CV, which combined and optimized three efficient computer recognition algorithms. The paper also proved the method's effectiveness in the experiment. Through real-time monitoring of the participant's facial signals, the intelligent system can identify two main antecedent causes of the EEG artifacts, participant's blinks and head movements, and annotate the artifacts' time segments in real time. In a semiconstrained environment where PEEGT is generally used, the intelligent computing method makes PEEGT break through the current application bottleneck limited by artifacts, which meets the needs of processing large-scale test data with low cost and simple operation demands. The method introduces a new perspective to neurophysiological measurements. It utilizes the algorithm with a readily available commercial camera instead of expensive laboratory equipment or/and high manual costs. In addition, it enlightens us on conducting large-scale testing in a semiconstrained environment outside the laboratory.

The innovation of introducing the CV method into neurophysiological measurements is noteworthy:

1. We proposed a new idea of detecting behavioral artifacts in EEG signals in real time. Therefore, the paper focuses on introducing the panorama of the method instead of the advantages of specific algorithms.
2. Most of the machine learning algorithms in neuroscience and behavioral science run offline, but real-time detection is the innovation that the paper emphasized; and scenarios described in the paper are not the same as offline artifact mark recognition and thus are not comparable.
3. We believe that interpretability is essential for a new method, and the algorithm black box is not conducive to trust and accept the innovation.

However, machine learning can effectively recognize blinking, head movement, and other behaviors in real time. Thus, in the specific recognition algorithms, we chose the method of logical judgment by feature points and achieved ideal detection results. Nevertheless, with the acceptance of this method and continuous optimization of the algorithm, intelligent methods will be applied on a larger scale. The accuracy of the CV method will be promoted, which is the fundamental advantage of the algorithm compared with the manual annotation.

On the other hand, the efficient and accurate annotation of artifacts caused by the subject's activities is the critical precondition step for intelligent artifacts removal. The algorithm can accurately capture the individual physiological activity differences among participants in the same actions, such as blinks, thanks to the CV method. Thus, the supervised machine learning algorithm can be based on the individual differences for more accurate individual artifact removal and correcting, which will greatly improve the accuracy of EEG artifact signal processing. That is the goal of the next stage of this paper. In the future, it will be critical for PEEGT to start large-scale commercial applications in more complex experimental environments, such as the engineering management, the effects of film and television programs, advertising research, information flow research, aroma cognition test, and game interaction test.

## Figures and Tables

**Table 1:** Five groups of experimental result.

	Blink left					Blink right					Slight head movement					Severe head movement				
	Detection	Wrong	Miss	TPR (%)	SPC (%)	Detection	Wrong	Miss	TPR (%)	SPC (%)	Detection	Wrong	Miss	TPR (%)	SPC (%)	Detection	Wrong	Miss	TPR (%)	SPC (%)
CV	108	24	2	97.67	97.05	104	21	3	96.51	97.42	35	4	2	93.94	99.54	9	0	0	100.00	100.00
Observer A	91	11	6	93.02	98.65	91	11	6	93.02	98.65	40	15	8	75.76	98.27	22	13	0	100.00	98.54
Observer B	89	9	6	93.02	98.89	89	9	6	93.02	98.89	61	36	8	75.75	95.85	15	6	0	100.00	99.33
Observer C	88	10	8	90.70	98.77	88	10	8	90.70	98.77	32	4	15	84.85	99.53	11	2	0	100.00	99.86
Reference value	86	—	—	—	—	86	—	—	—	—	33	—	—	—	—	9	—	—	—	—

TPR represents the sensitivity of the data: the rate of real blinks or head movements. SPC represents the specificity of the data: the rate of real nonblinking or non-head-movements. TPR = (detection-wrong)/(detection-wrong + miss) × 100%. SPC = (total-detection-miss)/(total-detection-miss + wrong) × 100%.

## Data Availability

Previously reported face feature selection data were used to support this study and are available at doi: 10.1109/CVPR.2014.241. These prior studies (and datasets) are cited at relevant places within the text as reference [29]. And the experiment data used to support the findings of this study are available from the corresponding author upon request.
